# Synthesis and Initial Evaluation of a Novel Fluorophore for Selective FMDV 3C Protease Detection

**DOI:** 10.3390/molecules25163599

**Published:** 2020-08-07

**Authors:** Samerah Malik, Alex Sinclair, Ali Ryan, Adam Le Gresley

**Affiliations:** Chemical and Pharmaceutical Sciences, SEC Faculty, Kingston University, Kingston-upon-Thames, London KT1 2EE, UK; k0613436@kingston.ac.uk (S.M.); alegresley1@hotmail.com (A.S.); ali.ryan@northumbria.ac.uk (A.R.)

**Keywords:** FMDV, 3C protease, fluorescence, veterinary testing

## Abstract

The development and evaluation of a Boc-AL(Boc)Q(Trt)-AMC fluorophore to detect 3C Protease, produced by Foot and Mouth Disease Virus (FMDV) is reported, with a view to a potential use as a rapid screen for FMDV infected livestock The peptide-linked conjugate fluorophore is evaluated in vitro for sensitivity, specificity, stability and rapidity and shows statistically significant increases in fluorescence when exposed to physiologically relevant concentrations of 3C Protease and selectivity when compared with other common proteases likely to be located, typically in the absence of FMDV. The stability of deprotected Boc-AL(Boc)Q(Trt)-AMC is reported as a limitation of this probe.

## 1. Introduction

Foot and Mouth Disease Virus (FMDV) is an extremely contagious pathogen and an outbreak caused by this virus can cause economic devastation, food insecurity, poverty and restrict food trade [1]. As a result, strategies to control, manage and prevent the spread of infection via early detection are a requirement. The World Organisation for Animal Health has published a manual of diagnostic tests and vaccines that defines FMDV diagnosis tests and states diagnosis can be achieved via virus isolation, detecting nucleic acid, viral antigen, virus specific or viral non-structural protein (NSP) antibodies irrespective of the vaccination status of the animal [2]. A number of lab-based diagnostic techniques are listed that can be used to detect viral pathogens and give a clinical diagnosis. Over the years, a range of these molecular biological tests have been modified to specifically detect FMDV: complement fixation test (CFT), virus neutralisation (VN), enzyme linked immunosorbent assay (ELISA) and polymerase chain reaction (PCR) [3,4,5].

Often, the delay in diagnosis is caused by the need of the infected sample to be transported to a high containment laboratory with certification to work with FMDV and requires skilled personnel using specialist equipment. Other limitations for consideration are: the need of the sample originating from a specific source to comply with the diagnostic technique—epithelial, blood and sputum, and the expenses involved in portable systems and the stability of reagents [6].

To date three main pen-side diagnostic technologies that have been reported include:

▪ Nucleic acid detection using RT-PCR;
▪ Antigen detection using different formats of lateral flow devices (LFD);
▪ LFD detection after isothermal amplification using primers of certain regions of the FMDV genome.

PCR is considered a powerful and sensitive technique, hence the development of portable PCR systems for FMDV detection; however, field testing made the limitations of the system apparent as it requires a precision thermo-cycling step that is carried out using expensive, fragile instrumentation that requires a vigorous decontamination protocol of the instrument to be followed after each site use [7]. In order to reduce the costs associated with the necessary cycling at different reaction temperatures for the portable PCR system, isothermal amplification strategies were explored in viral diagnostics. In 2000, Notomi et al. developed a molecular technique based on loop mediated isothermal amplification (LAMP), a technique widely used to detect viruses [8].

LFDs are rapid, deployable detection platforms; however, antibody/antigen based LFDs offer equivalent or less diagnostic sensitivity when compared to Ag-ELISA for certain serotypes, and can only be applied to the acute phase of FMD where samples collected contain high amounts of viral particles from vesicular or epithelial samples. These application requirements render the device useless in the incubation period and in the absence of obvious clinical signs, whereas isothermal-based assays allow for the evaluation of samples from various sample types: epithelial suspensions, serum and oesophageal–pharyngeal fluids [9,10].

A further complication with pen-side diagnostic kits is the need to maintain the integrity of the reagents, such as enzymes being utilized. To address this, methods have been developed to lyophilise reagents, making them stable under less optimal/harsher conditions, as seen in many areas affected by FMD. In 2015, Howson et al. reported the validity of the use of lyophilised reagents in field settings of East Africa (FMD endemic location) on RT-LAMP and RT-PCR assays, the group have reported no adverse effects on the performance of the assays [11]. Despite these advances, there is still scope for a rapid, point-of-decision-making test, which can be stored and deployed in the field easily [12]. In this paper, we take advantage of the presentation by FMDV of viral 3C protease (3C^Pro^).

3C^pro^ is a chymotrypsin-like cysteine protease. It is 213 amino acids long with a molecular weight of around 23kDa. The enzyme’s sequence is highly conserved, ~82–85% identical over all known serotypes without any cellular equivalents in host cells, and it is therefore a suitable target for our FMDV detection [13,14]. The enzyme cleaves the viral polypeptide chain and is vital for further processing of the sequence as it recognises and cleaves 10 of the 13 polypeptide junctions. It also interacts with a wide range of host proteins involved in the host’s immunity (Table 1), thereby successfully suppressing cellular immune responses. This shows that 3C^pro^ plays a critical role in viral pathogenesis.

3C^pro^ cleaves a large number of amino acid pairs during primary and secondary processing. These include: E/G, E/T, Q/K, Q/G, Q/T and Q/M, but the main sequence recognized and cleaved by 3C^Pro^ is H_2_N-Ala-Lys-Gln-OH.

The resolution of the atomic structure of 3C^pro^ FMDV revealed important structural features. The enzyme adopts a chymotrypsin like fold, as observed in most serine proteases with a conserved catalytic triad made up of Ser-His-Asp [19,20]. In an elegant example of this, Leatherbarrow et al. probed various oligopeptides as part of a DABCYL-EDANS FRET pair for use as a continuous 3C^Pro^ monitoring fluorophore [21]. Despite this, there has been little done to establish the selectivity of these fluorophores for 3C^Pro^ and there remains scope for the generation of new fluorogenic assays for use as rapid affordable field tests for FMDV [22].

## 2. Results

### 2.1. Synthesis

The H_2_N-Ala-Lys-Gln-OH tripeptide was coupled to AMC using peptide coupling conditions described in the Section 4. Initially, the synthesis of Boc-AK(Z)Q-AMC **1** proved capricious in terms of purification. As a result, a trityl protecting group was introduced to afford Boc-AL(Boc)Q(Trt)-AMC **2**.


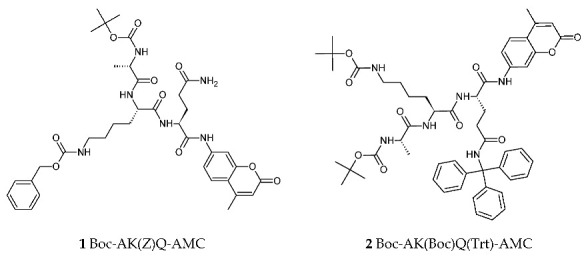


Introduction of the trityl group on to the glutamine’s side chain increased the stability and solubility of the compound, improving the handling of the compound during the purification process. However, the overall yield was still very low (Table 2), due to the reliance of the final coupling on a coupling reagent, a problem faced throughout the project.

Compounds **1** and **2** require acid treatment to deprotect the substrate ready for biochemical testing. In our hands, the unprotected tripeptide sequence was unstable; therefore, the ideal situation for the biochemical testing of this compound was to deprotect it in the presence of the target enzyme, to minimise any premature decomposition.

3C^pro^ was kindly provided by the Pirbright Institute in Surrey. Details of the mutations in the supplied 3C^pro^ strain are C95K and C142A., which are reported to improve solubility and proteolytic activity, respectively [23]. DSF was used to see if in situ trityl/Boc deprotection could be undertaken without detriment to 3C^pro^ activity. It was discovered that the level of TCA required for the deprotection of the substrate in situ would denature the protein, rendering it inactive (data not shown).

### 2.2. Cleavage by 3C^pro^—Sensitivity and Selectivity Testing

The activity of the 3C^pro^ in the presence of the deprotected Boc-AL(Boc)Q(Trt)-AMC **2** was evaluated by monitoring AMC fluorescence. Fluorescence readings were taken on the Infinite M200 PRO plate reader and the parameters were set to an excitation wavelength of 360 nm and emission wavelength of 455 nm in order to analyse the release of the fluorophore AMC.

**Figure 1:**
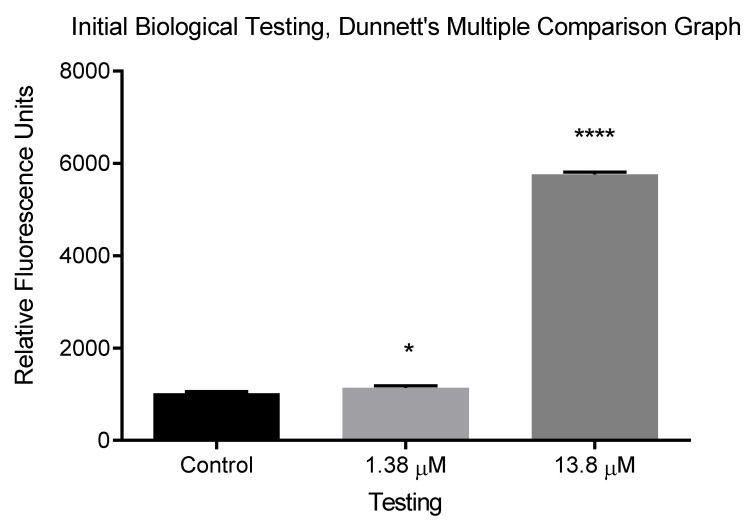
The graph represents the fluorescence data from biological testing after 10 min of incubation at 37 °C, one-way ANOVA data and Dunnett’s multiple comparison test results. Both these tests were run on Graphpad 6; the Dunnett’s multiple comparison data are represented using asterisks. (*): control, 2:[E] = 1.38 µM and 13.8 µM, respectively, [S] was controlled in all wells. The full details of concentrations and volumes used given in the appendix; Table 1. The error bars represent the standard deviation of replicates (*n* = 3).

Figure 1 shows the fluorescence data from the 3C^pro^ cleavage of the Boc-AL(Boc)Q(Trt)-AMC **2**, after 10 min of incubation at 37 °C, one-way ANOVA data and Dunnett’s multiple comparison test results. Both of these tests were run on Graphpad 6; the Dunnett’s multiple comparison data are represented using asterisks (* = *p* < 0.05, **** = *p* < 0.0001). The full details of concentrations and volumes used are in the Supporting Information. The error bars represent the standard deviation of replicates (*n* = 3) [24].

A significant statistical difference was seen using the one-way ANOVA test between the negative control and the testing wells at 13.8 μM enzyme (*p* < 0.0001), indicative of a significant change in fluorescence measurements. Further statistical analysis using the Dunnett’s multiple comparison test was performed to determine significance from the negative control and each testing well. This is represented in the graph shown in Figure 1. So, from the Dunnett’s multiple analysis, we found the higher [E] of 13.8 µM and 10 min incubation caused a more significant change in fluorescence when compared to the ten-fold less [E] of 1.38 µM with the negative control wells. Therefore, the higher concentration and volume of 3C^pro^ was used in further assay tests [23,24].

It is apparent that the fluorescence measurements at time = 0 were slowly increasing over time, for the stored deprotected substrate solutions [25]. An increase for the [S] at 100 µM from ~1000 RFU to ~2000 RFU was seen after 4 h in buffer solution (Supporting Information S2). This was thought to be due to the detection probe decomposing, releasing the free fluorophore and highlighting a major problem with stability, an issue faced throughout the synthesis and purification part of the project. Eventually, no change in fluorescence measurements was being recorded, indicating no substrate hydrolysis, as a possible result of the inevitable denaturation of the target enzyme or the decomposition of the unstable probe. Further evidence of cleavage in the presence of our target enzyme has been gained through increased fluorescence, substrate concentrations of 100 µM and 150 µM, this increase in fluorescence was found to be a significant change in comparison to the negative control using the one-way ANOVA statistical method (*p* < 0.005).

To further test the application of the detection probe, a series of experiments were designed to expose the conjugate to other proteases that may be found in a clinical sample taken from livestock infected with FMDV. The 3C^pro^ enzyme is reported to have properties characteristic of cysteine and serine proteases; therefore, the following commonly occurring serine type proteases were selected to test against: chymotrypsin, thrombin and trypsin. To specifically test the amino acid sequence attached to the fluorophore, another enzyme was selected—Tobacco Etch Virus (TEV)—as this is a related cysteine protease with a Q/G and Q/S selectivity similar to that mentioned for the 3C protease. This illustrates the importance of the protease when evaluating selectivity. The consensus sequence for TEV is Glu-Asn-Leu-Tyr-Phe-Gln.

**Figure 2:**
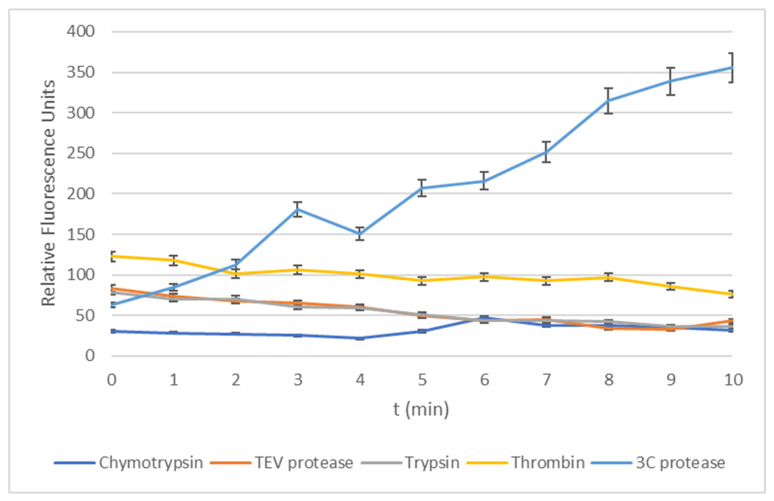
Graph of normalised data between 0–400 to show comparison of all enzymes tested with substrate **2** with 3C^pro^. Concentrations for enzymes between 13.8 µM–15 µM and substrate 100 µM. Errors bars show 5% deviation.

From Figure 2, it is apparent that substrate **2** shows selectivity to the 3C^pro^ enzyme over commonly occurring enzymes in clinical samples. The TEV protease was found to have no significant change in fluorescence measurements recorded in comparison to the negative control using the one-way ANOVA statistical method (*p* > 0.05). Furthermore, the duration of time fluorescence measurement was chosen to be the same length as the assay experiments run with the target enzyme 3C^pro^ reported earlier as, in the presence of the 3C^pro^, fluorescence would be generated more rapidly and, therefore, a delayed increase in fluorescence would be indicative of the breakdown of the conjugate via other routes.

## 3. Discussion

From the biochemical testing, the proof of breakdown was gained from our detection probe as a consequence of our target enzyme’s activity.

Testing using a range of common proteases shows that the substrate was selectively cleaved by 3C^pro^, but not by chymotrypsin, thrombin, trypsin and the TEV protease. From the results obtained, it can be concluded that our detection probe is recognised by the 3C^pro^ and the enzyme is capable of recognising and processing shorter fragments of peptides, previously reported not to be the case [26].

Whilst substrate **2** is stable, upon removal of the trityl group, it appears to decompose in solution. Despite this, we can quite confidently conclude the cleavage of substrate 2, after removal of the trityl groups, was enzyme-assisted and not due to spontaneous decomposition as the measurements recorded from the control wells (enzyme-absent well) remained constant. We also observed the selectivity of our probe for 3C^Pro^ in comparison with other proteases, suggesting the evolution of fluorescence is enzyme-linked.

However, in solution, the deprotected detection probe was found to have a short shelf-life in comparison to a commercially available BocValProArg-AMC. Although the imminent need for a rapid detection probe has been highlighted in this paper and the recognition of the smaller peptide fragment by 3C^pro^ has been proven, further work on the stability of the deprotected **2** could potentially provide a more commercially viable detection probe. Immediate immobilization of the BocValProArg-AMC fragment is a possibility.

The AKQ sequence, appears prone to hydrolysis in solution; however, is otherwise stable as a dry solid. From a practical perspective, this may be a limitation, owing to the need to prepare a solution thereof prior to use, however the sensitivity and selectivity of substrate ***2*** after the removal of the trityl group is compelling and, in this manuscript, we have proved the principle of a selective fluorescent probe, which could be applied as a rapid point-of-decision-making test for FMDV in the field. The authors acknowledge that such an application would demand a stable vehicle for the probe.

## 4. Materials and Methods

### 4.1. Synthesis

NMR was recorded using a Bruker (Coventry, UK) Avance III 400 two channel FT-NMR spectrometer and the Bruiker Avance III 600 three channel FT-NMR spectrometer. ^1^H NMR spectra were recorded at either 400 or 600 MHz and ^13^C NMR spectra were recorded at 100 MHz. ^19^F NMR spectra were recorded at 376 MHz. Chemical shifts were referenced to the solvent used and noted in the experimental. IR (infrared) was recorded using a Perkin Elmer Spectrum (Buckinghamshire, UK) 100 FT-IR spectrometer. GC/MS was recorded on an Agilent Technologies 5973 mass selective detector, 6890 N Network GC system. HR-MS and elemental analysis results were obtained via Medac Ltd. (Woking, UK).

TLC analysis was carried out on silica, aluminium oxide, reverse phase silica coated plates and were visualised by a single method or a combination of the following methods: (a) viewing under UV at 254 nm; (b) viewing under UV at 365 nm; (c) exposure to a ninhydrin solution, containing 2 g of ninhydrin in 60 mL of ethanol; (d) exposure to a vanillin solution, containing 6g of vanillin, 250 mL of ethanol and 1.5 mL of 12M aqueous sulphuric acid.

Preparative TLC was carried out on normal phase silica-based plates; the mobile phase solvent mix is described in ratios in the procedure details. Manual columns were run using normal phase silica of particle size 250–500 µm and 35–60 mesh. Representative NMR and HR-MS data for substrate **2** are in Supporting Information S4.

#### 4.1.1. Synthesis of **1**

Under a nitrogen atmosphere, BocAlaLys(Z)OH (65mg, 0.14 mmol, 1.5 eq.), NH_2_GlnAMC (29 mg, 0.96 mmol, 1 eq.) and HATU (72 mg, 0.19 mmol, 2eq.) were dissolved in 1.5 mL of anhydrous DMF. To this solution the base DIEA (5.68 g, 80 µL, 5 eq.) was added. The resultant yellow solution was left at 50 °C for a week. The reaction progression was monitored via TLC. The reaction was quenched with 2 mL of water and extracted with 6 mL ethyl acetate and further washed with 3 × 2 mL saturated lithium chloride solution and dried over Na_2_SO_4_. The reaction mixture was concentrated under reduced pressure and placed on the high vacuum. The crude mixture was purified using preparative TLC (6.5% MeOH:DCM) (3mg, 0.4%),^1^H NMR (400 MHz, methanol-*d*_4_) δ ppm 1.26–1.35 (m, 1 H), 1.41–1.46 (m, 9 H), 1.47–1.51 (m, 1 H), 1.51–1.52 (m, 1 H), 1.52–1.54 (m, 1 H), 1.88–1.90 (m, 1 H), 2.43–2.48 (m, 1 H), 2.69–2.73 (m, 1 H), 2.91–2.93 (m, 1 H), 3.09–3.16 (m, 1 H), 3.34–3.36 (m, 1 H), 3.45–3.50 (m, 1 H), 3.62–3.65 (m, 1 H), 5.02–5.07 (m, 1 H), 6.21–6.26 (m, 1 H), 7.25–7.38 (m, 1 H), 7.67–7.76 (m, 3 H), 7.85–7.87 (m, 1 H) and 7.90–7.95 (m, 1 H). Electrospray, Time of flight, Mass Spectrometry (ES TOF MS) found 737.35, requires 736.8 for C_37_H_48_N_6_O_10_. The molecular formula verified by elemental analysis: C, 60.01; H, 6.87; N, 11.37; O, 21.70.

#### 4.1.2. Synthesis of **2**

Under a nitrogen atmosphere, BocAlaLys(Boc)Gln(Trt)OH (50 mg, 0.0634 mmol, 1 eq.), AMC (11 mg, 0.0635 mmol, 1 eq.) and HATU (48 mg, 0.127 mmol, 2 eq.) were dissolved in 5 mL of anhydrous DMF. To this solution, the base DIEA (41 mg, 55 µL, 5 eq.) was added. The resultant yellow solution was left at 50 °C for 3 nights. The reaction progression was monitored via TLC. The reaction was quenched with 10 mL of water and extracted with 3 × 10 mL ethyl acetate and further washed with 3 × 8 mL saturated lithium chloride solution and dried over Na_2_SO_4_. The reaction mixture was concentrated under reduced pressure and placed on the high vacuum. The crude mixture was purified using preparative TLC with solvent conditions: 5%/95% MeOH:DCM to give BocAlaLys(Boc)Gln(Trt)AMC as a clear oil (10 mg, 15.7%). ^1^H NMR (400 MHz, methanol-*d*_4_) δ ppm 1.05–1.22 (m, 3 H), 1.23–1.35 (m, 4 H), 1.37–1.53 (m, 18 H), 2.81 (s, 1 H), 2.86 (d, *J* = 0.75 Hz, 1 H), 2.96–3.08 (m, 2 H), 3.69 (d, *J* = 6.78 Hz, 1 H), 3.81–4.17 (m, 1 H), 4.34–4.51 (m, 1 H), 4.58–4.67 (m, 1 H), 4.68–4.80 (m, 1 H), 6.22–6.25 (m, 1 H), 7.13–7.33 (m, 15 H). ^13^ C NMR (100 MHz, methanol-*d*_4_) δ ppm, 16.61, 17.31, 18.43, 19.91, 23.10, 27.45, 28.12, 28.41, 29.93, 30.35, 30.83, 31.31, 32.6, 33.24, 33.33, 36.46, 37.10, 38.35, 40.23, 49.05, 49.20, 51.56, 54.27, 111.13, 117.25, 121.35, 122.05, 127.04, 128.18, 128.21, 133.5. The mass, confirmed by Electrospray, Time of flight, Mass Spectrometry (ES TOF MS) was found to be 967.4595 C_53_H_64_N_6_O_10_Na, requiring 945 for C_53_H_64_N_6_O_10_. The molecular formula was verified by elemental analysis: C, 67.31; H, 6.87; N, 8.81; O, 16.70.

### 4.2. SDS-PAGE

3C^pro^ was kindly provided by the Pirbright Institute in Surrey (volume of 100 µL at concentration of 13.8 µM). Details of the mutations in the supplied 3C^pro^ strain are C95K and C142A. These substitutions are reported to improve solubility and proteolytic activity, respectively. The enzyme was stored at −80 °C on arrival and thawed to room temperature for biological assays.

### 4.3. DSF

Following an adapted protocol reported by Niesen et al., pepsin (from porcine stomach mucosa Sigma-Aldrich, Dorset, UK) was diluted to 64 µg/mL in PBS pH 7.4, 0.5 mM EDTA and 5× Sypro Orange (Oxoid). The fluorescence was measured using an MX3005p qRT-PCR (Stratagene) and the temperature was increased from 25 °C to 95 °C at an increment of 1 °C /minute.

Spectral properties were used for the detection of Sypro Orange: excitation wavelength at 300/472 nm and emission wavelength at 570 nm. Data analysis was completed using software tools obtained from the Structural Genomics Consortium Oxford (ftp://ftp.sgc.ox.ac.uk/pub/biophysics). The raw DSF data were fitted to the Maxwell–Boltzmann distribution in Graphpad Prism 6 to determine the T_M_ value.

### 4.4. Enzymatic Fluorescence Assay

All enzyme measurements were performed at room temperature in white round-bottomed plates using an Infinite M200 plate reader (Tecan). To test for the turnover of the substrate by 3C protease, 100 µM of deprotected AlaLysGlnAMC substrate was mixed with 13.8 µM or 1.38 µM enzyme in a final reaction volume of 100 µL PBS 5% DMSO. Proteolytic cleavage was measured via increased fluorescence intensity (ex 365, em 450). To test for the selectivity of cleavage, all other enzymes were tested under identical experimental conditions.

## Figures and Tables

**Table 1:** Host-cell proteins cleaved by 3C^pro^.

Host Cell Protein	Function of Protein
eIF4G, eIF4A1 [15]	Eukaryotic translation initiation factors
H3 [16]	Histone
NEMO [17]	NF-kappa-B an essential modulator for IFN α/β responses
Sam68 [18]	Sequence-specific RNA binding protein that regulates alternative splicing

**Table 2:** Fluorogenic substrates successfully isolated and their overall percentage yields.

Fluorogenic Substrate Successfully Isolated	Overall Yield
Boc AL(Z)QAMC, 1	0.4%
Boc AL(Boc)Q(Trt)AMC, 2	15.7%

## Supplementary Materials

The following are available online, S1 DSF; S2 Dose Response of 3C^pro^ to Substrate 2; S3 SDS-PAGE Conditions; S4 Characterisation data for BocAlaLys(Boc)Gln(Trt)AMC, 2; Figure S1. Measuring the effects of TCA on the thermal melt curve of the common enzyme pepsin. The changing percentages of acid are: A (control), 0% TCA; B, 1% TCA; C, 0.5% TCA; D, 0.25% TCA; Figure S2. Performance testing results represented as fluorescence generation in the presence of the target enzyme, 3C^pro^, [E] = 13.8 µM. The error bars represent the standard deviation of replicates (*n* = 3); Table S1. Biological materials for selectivity testing. All enzymes were stored at −5 to −20°C and thawed to room temperature before running biological assays.
