# Supplementary material for: Synthesis and Initial Evaluation of a Novel Fluorophore for Selective FMDV 3C Protease Detection
Source: Molecules. 2020 Aug 7;25(16):3599. doi: 10.3390/molecules25163599 (PMC7465021; doi:10.3390/molecules25163599)
Supplement: Supplementary file 1 [file molecules-25-03599-s001.pdf]

Synthesis and initial evaluation of a novel fluorophore for selective FMDV 3C Protease detection

Samerah Malik, Alex Sinclair Ali Ryan and Adam Le Gresley*

Supporting Information

S1 DSF
In order to define the parameters needed for the unstable detection probe in the enzymatic assay, Differential Scanning Fluorimetry (DSF) was employed as a technique to monitor the effects of the percentage Trichloro acetic acid (TCA) on a related protease – pepsin, to establish a balance between maintaining the protein's structure whilst providing a suitable environment needed for the deprotection of the protecting groups attached to the detection probe. These conditions would allow the deprotection of the detection probe to occur in the presence of its target enzyme, therefore eliminating risk of the probe decomposing before having contact with its target enzyme.[12]

Thermal stability of the protein was tested by adding the dye (Sypro Orange) which binds to the hydrophobic parts of the enzyme as it denatures with increasing temperature. The intensity of the fluorescence detected by DSF is directly proportional to how effectively the dye is bound therefore the temperature at which the protein denatures is ascertained by the increase in fluorescence.

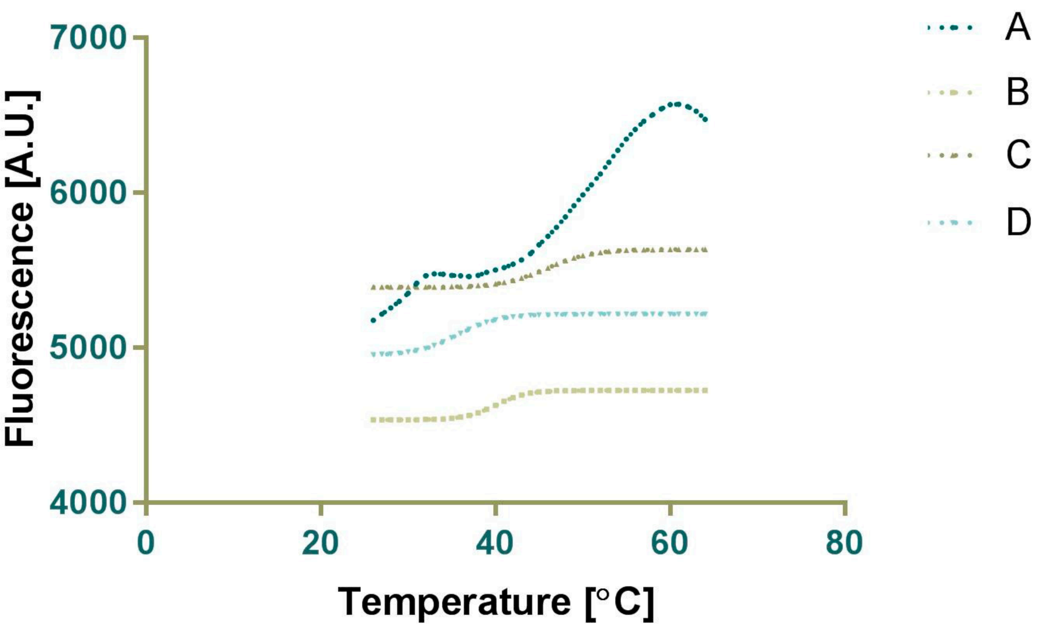

Figure S1. Measuring the effects of TCA on the thermal melt curve of the common enzyme pepsin. The changing percentages of acid are: A (control), 0% TCA; B, 1% TCA; C, 0.5% TCA; D, 0.25% TCA.

Figure S1 shows changes in thermal stability of the enzyme pepsin and emission of fluorescence by Sypro Orange. The data shows a significant reduction in stability of the protein in the presence of all tested TCA levels therefore T_M value: 50°C ±0.5°C could only be determined from curve A- the acid free sample. These results show the addition of an acid to the detection based assay would not be suitable as the enzyme is significantly destabilised even at the lowest 0.2% TCA tested, decreasing the acid percentage further would not be sufficient for deprotecting the Boc and trityl protecting groups

attached to the amino acid sequence of our detection probe. Therefore the deprotection step was designed to precede the enzymatic assay.

S2 Dose Response of 3C^Pro to deprotected **2**

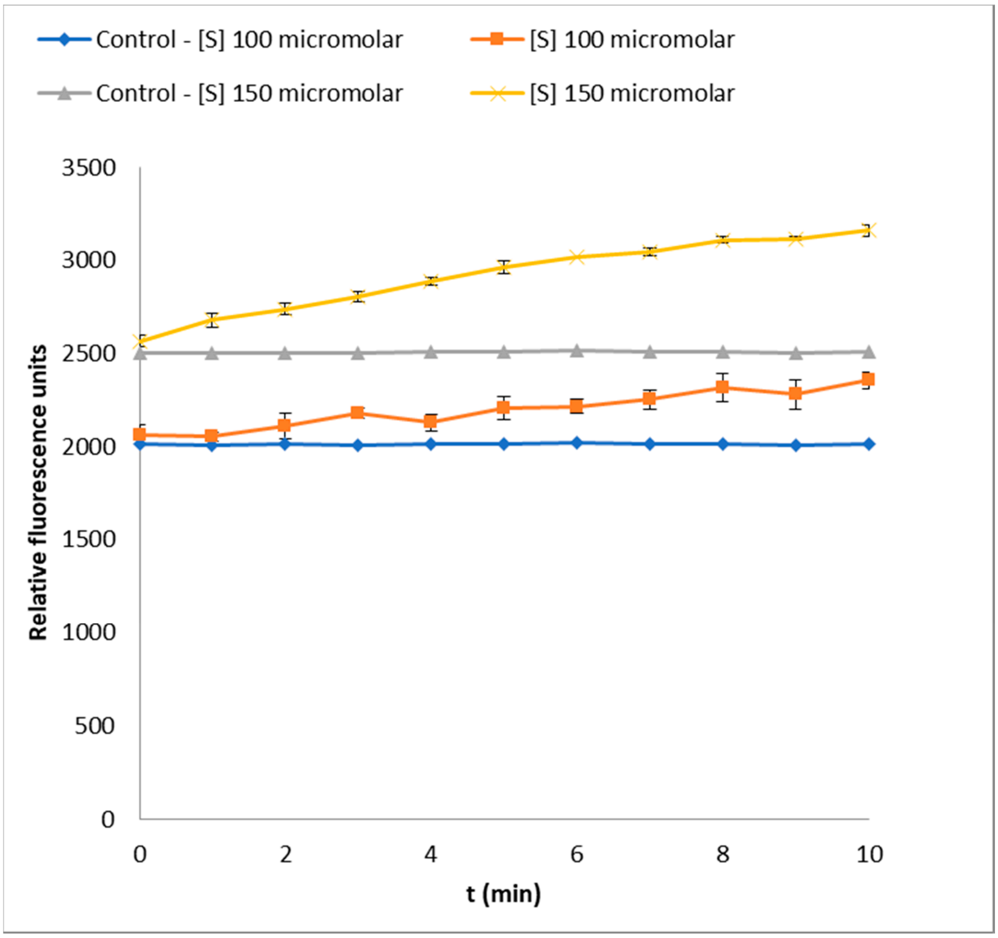

Figure S2. Performance testing results represented as fluorescence generation in the presence of the target enzyme, $3C^{Pro}$, [E] = 13.8μM. The error bars represent the standard deviation of replicates (n= 3).

Enzyme	Extracted from
Trypsin type 1	Bovine pancreas
Chymotrypsin type 2	Bovine pancreas
Thrombin	Bovine plasma
TEV protease	Recombinant expressed in *Escherichia coli*

Table S1. Biological materials for selectivity testing**.** All enzymes were stored at -5 to -20°C and thawed to room temperature before running biological assays.

S3 SDS-PAGE Conditions

To verify the purity of the enzyme sample we received from the Pirbright Institute, the technique SDS-PAGE was used. The gel was run in a MiniProtean Tetra system (BIO-RAD) with a SDS running buffer (National Diagnostics) (Table 6). The acrylamide gels consisted of a resolving gel and a stacking gel of

concentrations 12% w/v and 6% w/v respectively. The protein sample was prepared by adding a loading dye in a 1:1 volume ratio The sample mix was denatured by heating at 95° for 5 minutes to ensure protein conformation had no effect on the protein migration through the gel and the distance travelled can then solely be attributed to the molecular weight. 20 μL of the protein sample/ loading dye mix was added to the well and run alongside the molecular weight marker ECL Full-Range Rainbow molecular weight marker (Amersham) in a neighbouring well. Electrophoresis was run under 180V for 60 minutes (BIO-RAD PowerPak 300). The gel was stained for 30 minutes in a 1% w/v Coomassie Blue solution at room temperature under gentle shaking conditions: 90 RPM. The gel was then rinsed with water and de-stained using 10% v/v ethanol, 10%v/v acetic acid for 17 hours at room temperature under gentle shaking conditions: 90 RPM. The destained gel imaged using a Geldoc XR+ (BIORAD).

S4 Characterisation data for BocAlaLys(Boc)Gln(Trt)AMC, **2**

C53 H64 N6 O10

945.109g/mol

1H NMR for BocAlaLys(Boc)Gln(Trt)AMC

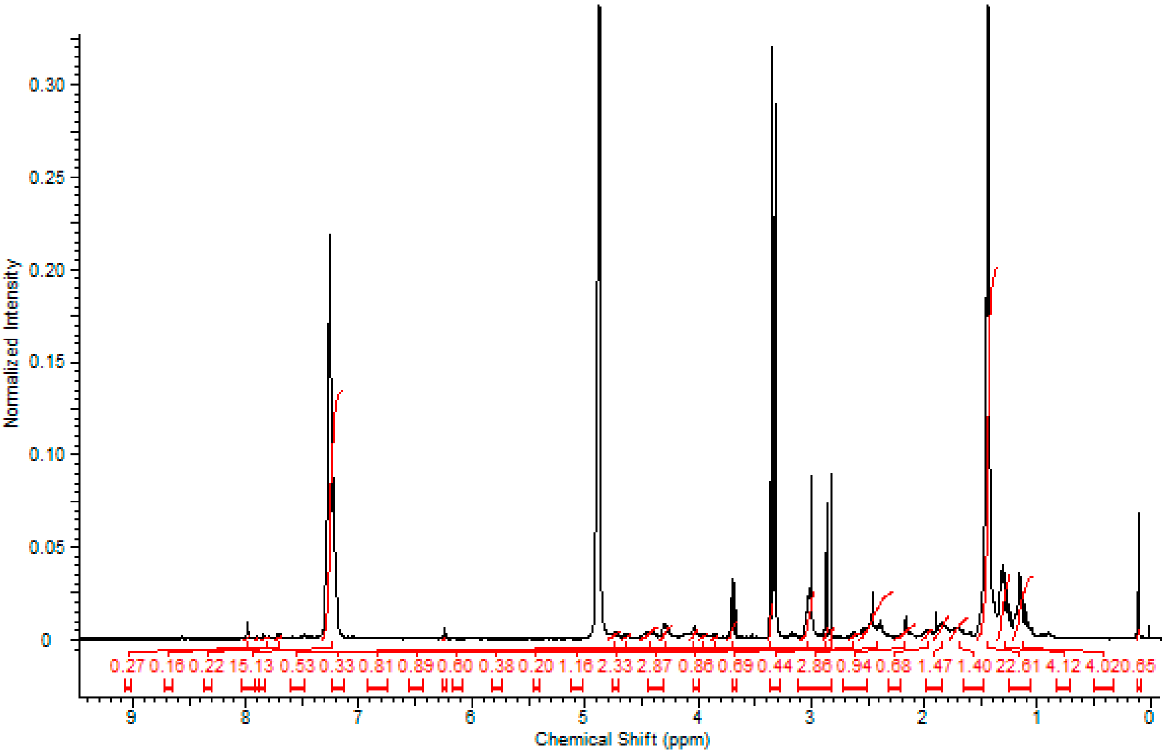

2D HSQC for BocAlaLys(Boc)Gln(Trt)AMC

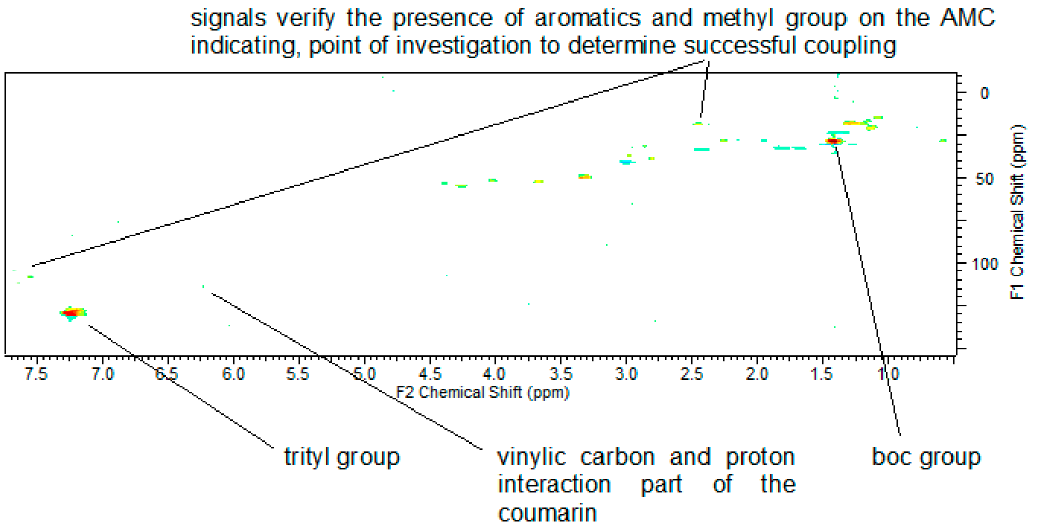

The ^{13}C assignment has been reported using the HSQC projections of the molecule because with reduced overall yield and large number of carbons in the molecule, a good C spectral analysis would only have been possible with an extended run > 18 hrs. From previous NMR experiments of amino-acid coumarin based compounds it is known that this prolonged NMR experimentation isn't suitable for decomposition prone compounds such as this unstable fluorogenic substrate, hence the shorter HSQC experiment was used to express the ^{13}C assignments.

^{13}C NMR (100 MHz, MeOD) ppm,16.61, 17.31, 18.43, 19.91, 23.10, 27.45, 28.12, 28.41, 29.93, 30.35, 30.83, 31.31, 32.6, 33.24, 33.33, 36.46, 37.10, 38.35, 40.23, 49.05, 49.20, 51.56, 54.27, 111.13, 117.25, 121.35, 122.05, 127.04, 128.18, 128.21, 133.5.

HR-MS

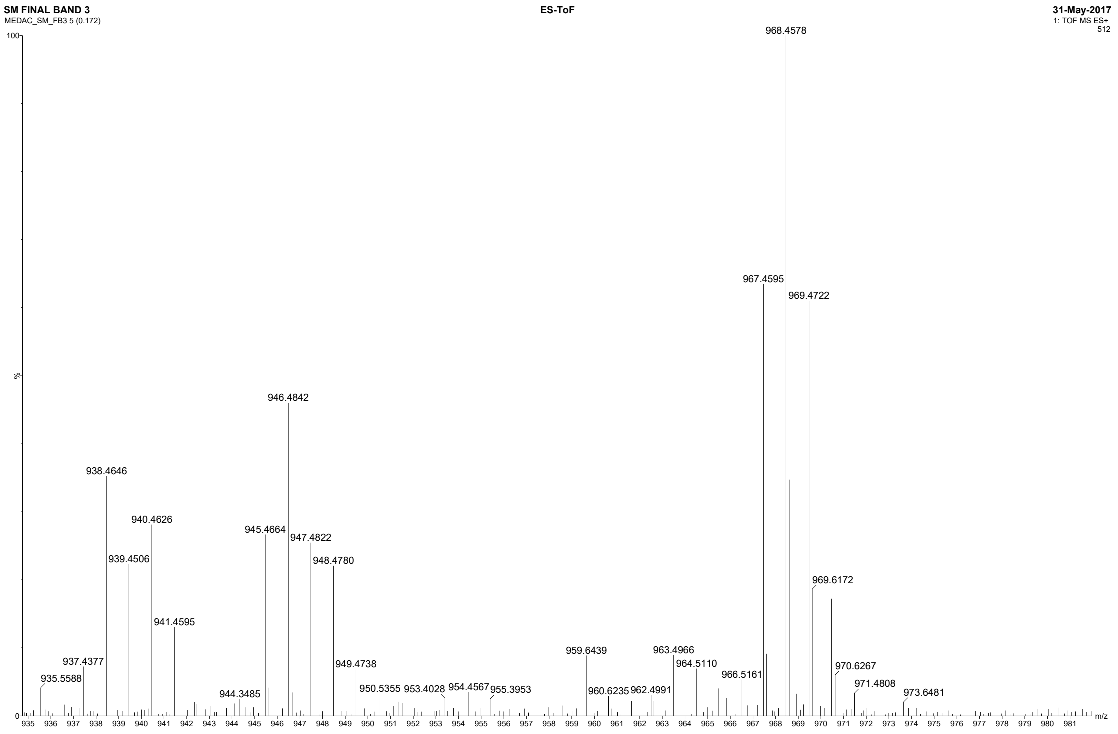

Mass	Calc. Mass	mDa	PPM	DBE	i-FIT	i-FIT

(Norm) Calc. Formula

967.4595	967.4582	1.3	1.3	24.5	71.2	0.0

C53 H64 N6 O10 Na

Elements Used:

C: 53-53 H: 0-200 N: 0-10 O: 0-10

Formula calculated:

C53 H64 N6 O10
